# 

**Published:** 1842-04-16

**Authors:** 


### MEDICAL EXAMINER,

EDITED BY

*J. B. BIDDLE, MD., & W. W. GERHARD, M. D.*

VOL. I.] APRIL 16, 1842. [No. 16.

